# Construct Validity and Internal Consistency of the Italian Version of the PedsQL^TM^ 4.0 Generic Core Scale and PedsQL^TM^ 3.0 Cerebral Palsy Module

**DOI:** 10.3390/children12060749

**Published:** 2025-06-09

**Authors:** Ilaria Pedrinelli, Sofia Biagi, Domenico Marco Romeo, Elisa Musto, Valeria Fagiani, Martina Lanza, Erika Guastafierro, Alice Colombo, Andrea Giordano, Cristina Montomoli, Cristiana Rezzani, Tiziana Casalino, Eugenio Mercuri, Daria Riva, Matilde Leonardi, Giovanni Baranello, Emanuela Pagliano

**Affiliations:** 1 Pediatric Neuroscience Department, Fondazione IRCCS Istituto Neurologico Carlo Besta, 20133 Milan, Italy; ilaria.pedrinelli@istituto-besta.it (I.P.); sofia.biagi@istituto-besta.it (S.B.); tiziana.casalino@istituto-besta.it (T.C.); daria.riva@istituto-besta.it (D.R.); 2 Paediatric Neurology Unit, Catholic University, 00168 Rome, Italy; domenicomarco.romeo@policlinicogemelli.it (D.M.R.); elisa.musto@fondazionesantalucia.it (E.M.); eugeniomaria.mercuri@policlinicogemelli.it (E.M.); 3 Emergency Department, Fondazione IRCCS Ca’ Granda Ospedale Maggiore Policlinico Milan, 20122 Milan, Italy; valeria.fagiani@unimi.it; 4 Neurology, Public Health and Disability Unit, Fondazione IRCCS Istituto Neurologico Carlo Besta, 20133 Milan, Italy; martina.lanza@istituto-besta.it (M.L.); erika.guastafierro@istituto-besta.it (E.G.); alice.colombo@istituto-besta.it (A.C.); andrea.giordano@istituto-besta.it (A.G.); matilde.leonardi@istituto-besta.it (M.L.); 5 Department of Public Health Neuroscience, Experimental and Forensic Medicine, University of Pavia, 27100 Pavia, Italy; cristina.montomoli@unipv.it (C.M.); cristiana.rezzani@gmail.com (C.R.); 6 Paediatric Neurology/Neuromuscular Disorders, University College London, Great Ormond Street Institute of Child Health, Great Ormond Street Hospital NHS Foundation Trust, London WC1N 1EH, UK; g.baranello@ucl.ac.uk

**Keywords:** Cerebral Palsy, children, health-related quality of life, Italian population, PedsQL

## Abstract

Background: Health-related quality of life (HRQoL) has emerged as a meaningful outcome measure in clinical trials and healthcare interventions in children with cerebral palsy (CwCP). We assessed the construct validity and internal consistency of the Italian version of the Paediatric QoL inventory (PedsQL^TM^) 4.0 Generic Core Scales (GCS) and PedsQL^TM^ 3.0 Cerebral Palsy Module (CPM). Methods: A total of 125 CwCP and their parents were enrolled. Participants completed both the GCS and the CPM modules, and the results were compared to those of a sample of 121 healthy peers and their parents. The dimensionality of the two modules was assessed through exploratory factor analysis. Construct validity was assessed by a known-groups method evaluating the differences between CwCP and healthy sample. Results: Only a few GCS subscales were unidimensional, while all CPM subscales proved to be unidimensional, except for the Speech and Communication subscales of child self-reports. GCS internal consistency was good for all subscales of the parent proxy-reports, as well as for the Physical Activities and Psychosocial Health subscales of child self-reports. CPM internal consistency was good for both parent proxy-reports and—with a few exceptions—child self-reports. As for the PedsQL^TM^ validity, the GCS proved effective in discriminating between CwCP and healthy participants; the CPM showed a significant association between lower neurofunctional abilities and lower HRQoL. Parent–child concordance shows that child self-report scores were always higher than the those of the proxy-reports for both the GCS and CPM modules. Conclusions: The present study confirms the internal consistency and construct validity of the Italian version of both PedsQL^TM^ modules. In CwCP, greater functional disability resulted in lower HRQoL scores, and there was significant discrepancy between the parent and child ratings.

## 1. Introduction

Cerebral palsy (CP) represents one of the leading causes of neurological disability in the paediatric population, affecting 1.6 per 1000 live births, with consequences that profoundly affect individuals and their family lives [1]. CP is defined and classified as a complex disorder that limits movement and affects posture, sensation, perception, cognition, and behaviour. Thus, physical symptoms should not be the sole focus of treatment; there are other key aspects, such as independence in activities of daily living and quality of life (QoL), that should be considered.

The International Classification of Functioning, Disability and Health (ICF), has emphasised the need for family-centred models of healthcare and an ecological approach to treatment that seek to ensure that children with CP (CwCP) and their families have a satisfying QoL [2]. Moreover, the past decade has evidenced a large increase in the development and use of paediatric Health-Related Quality of Life (HRQoL) measures to improve patient health and well-being and determine the value of healthcare services [3,4].

One definition of HRQoL is “an individual’s perception of various aspects of his/her life that he/she believes are affected by a particular medical condition or treatment” [5]. Self- or parent-perceived HRQoL is a key target in rehabilitation practice and has emerged as a significant outcome measure in clinical studies and healthcare interventions [6,7]. Indeed, HRQoL measures are particularly useful in assessing the effectiveness of different treatment approaches, guiding physicians in the clinical decision-making process. In addition, HRQoL measures provide an opportunity for parents and children to actively participate in therapeutic choices, according to the family-centred model of healthcare [4]. Generic HRQoL measures are usually multidimensional and include at least the physical, psychological (including emotional and cognitive), and social dimensions of health, as outlined by World Health Organisation. While disease-specific HRQoL measures can improve measurement sensitivity for health domains related to a particular chronic condition, the use of generic HRQoL measures allow for comparisons across chronic conditions and benchmarking with healthy population samples [8]. From the need to use more relevant and meaningful outcome measures for patients and their caregivers, HRQoL assessment tools have been developed [9,10].

Although HRQoL is subjective and should be self-reported, it is often difficult or impossible to obtain such reports in populations with severe CP because of the accompanying cognitive and communication impairments [11]. Despite the literature offering an increasing number of valid generic and disease-specific HRQoL measures, these are still not homogeneous and are rarely specifically designed for CwCP [3,12].

An important HRQoL measure, developed and validated by Varni and colleagues [13], is the Paediatric Quality of Life Inventory (PedsQL^TM^) Measurement Model, which was developed with a multidimensional approach, and which allows for the integration of a generic and a disease-specific evaluation through distinct modules, namely, the PedsQL^TM^ 4.0 Generic Core Scale (GCS) and the PedsQL^TM^ 3.0 Cerebral Palsy Module (CPM), designed to assess HRQoL dimensions specific to CP. PedsQL^TM^ modules offer both the opportunity to compare self-reports and parent proxy-reports and the possibility to cover a wide range of ages within the paediatric population (from 2 to 18 years), allowing for longitudinal analyses on account of the dynamic nature of the construct.

This study is the first to validate the Italian version of the PedsQL™ in children with cerebral palsy, comparing their results to those of a control group of healthy peers. Furthermore, it highlights the importance of using a feasible, reliable, and validated questionnaire tailored towards Italian children with cerebral palsy within the framework of a family-centred approach.

The aims of the present study were to investigate (i) the internal consistency and construct validity of the Italian version of PedsQL^TM^ GCS and CP modules; and (ii) the differences in concordance between children’s self-report and parent proxy-reports. We hypothesised that the Italian version of the PedsQL^TM^ GCS and CP modules would show reliability and construct validity equal to those of the original English version [14]. We also hypothesised that a direct correlation between HRQoL and functional status could be observed in a wide sample of Italian CwCP, and that significant differences could be found between child self-report and parent proxy-report.

## 2. Materials and Methods

### 2.1. Study Design and Population

**Figure 1:**
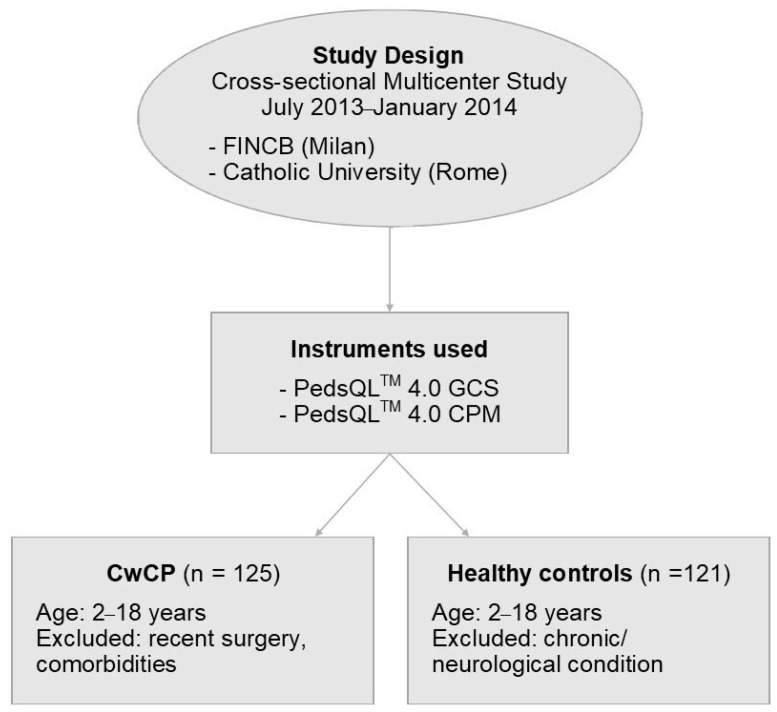
Flow diagram reporting study design, sample, and instruments used. CwCP, children with cerebral palsy; CPM, Cerebral Palsy Module; FINCB, Fondazione Istituto Neurologico Carlo Besta; GCS, Gross Core Scales; PedsQL, paediatric QoL inventory.

In this multicentre cross-sectional study, 125 CwCP and 121 healthy peers, along with their parents, were enrolled. All eligible children, aged between 2 and 18 years, and their parents, living in Milan and Rome filled, filled out both the CPM and the GCS modules (Figure 1).

#### 2.1.1. CwCP Sample

Between July 2013 and January 2014, all children consecutively assessed for routine clinical activity at the Developmental Neurology Unit of the Fondazione IRCCS Istituto C. Besta (FINCB, Milan, Italy) and at the Child Neurology Unit of Catholic University (Rome, Italy) were recruited as part of a collaborative prospective project on families of CwCP. The protocol was approved by the Ethics Committees of the two centres, and the study was conducted in accordance with the Declaration of Helsinki. Inclusion criteria were an age between 2 and 18 years, confirmed diagnosis of CP [11], the absence of surgical interventions within the previous 3 months, and the absence of other comorbid diagnoses to prevent other medical conditions from interfering with the HRQoL assessment. All children were classified according to the Gross Motor Function Classification System (GMFCS) [15] and, for those older than 4, according to the Manual Ability Classification System (MACS) [16]. All children also underwent structured cognitive assessment according to age via the Griffiths Mental Development Scale—Extended Revised (GMDS–ER) [17], the Wechsler Preschool and Manuscript Primary Scale of Intelligence III (WIPPSI-III) [18], and the Wechsler Intelligence Scale for Children (WISC III) [19]. After receiving written informed consent from parents and children aged 8–18 years, the questionnaires were administered separately to children and their parents, with research personnel assisting them in a supervisory role. Children aged 5 to 7 years, as well as those who were unable to read/write because of physical/cognitive impairment, were verbally administered the questionnaires and could answer non-verbally (i.e., through nodding/pointing). Moreover, for children aged 2 to 4 years, informed consent was not required from the children themselves as only the parent proxy questionnaires were administered in this age window.

When children were completely incapable of self-reporting because of particularly severe conditions (e.g., tetraplegia), only the parental version was administered. A family information form, including sociodemographic and clinical variables (gender, age, family status, level of education of parents, presence of epilepsy, access to rehabilitation), was also collected. All data were collected anonymously, and confidentiality of personal data was guaranteed to all participants.

#### 2.1.2. Healthy Children Sample

Healthy children and their parents were recruited from nursery schools and primary and secondary schools close to the enrolling centres in Milan and Rome. The healthy sample included children aged 2 to 18 years with no neuropsychological disorders or severe chronic conditions in the last 6 months, as well as their parents. After receiving written informed consent from scholastic educational institutions and families, research personnel distributed the questionnaires in each classroom and assisted the children in completing them. Parents filled out the PedsQL^TM^ modules and the family information form separately at home and returned them to the school. All data were collected anonymously, and privacy was guaranteed.

### 2.2. Measures

#### 2.2.1. PedsQL^TM^ 4.0 GCS

The 23-item PedsQL^TM^ 4.0 GCS includes 4 subscales: (1) Physical Functioning; (2) Emotional Functioning; (3) Social Functioning; and (4) School Functioning. In line with the indications of Varni and colleagues [13], we calculated the Psychosocial Health summary score as the sum of all items divided by the number of items answered in the Emotional Functioning, Social Functioning, and School Functioning scales. A 5-point Likert response scale ranging from 0 (never a problem) to 4 (almost always a problem) was used across child self-reports and proxy-reports. Items were scored in a reverse order and linearly transformed to a 0–100 scale (0 = 100; 1 = 75; 2 = 50; 3 = 25, 4 = 0). We used the Italian version of the PedsQL^TM^ 4.0.

#### 2.2.2. PedsQLTM 3.0 CPM

The 35-item PedsQL^TM^ 3.0 CPM includes seven subscales: (1) Daily Activities; (2) School Activities; (3) Movement and Balance; (4) Pain and Hurt; (5) Fatigue; (6) Eating Activities; (7) Speech and Communication. The School Activities and Speech and Communication subscales are not reported in the parental version of the toddlers’ (aged 2–4 years old) form. Additionally, the Daily Activities and Eating Activities subscales include fewer items. The format, instructions, Likert response scale, and scoring method are the same as for the PedsQL^TM^ 4.0 GCS, with higher scores indicating better HRQoL (i.e., fewer symptoms/problems). The Italian version of this module was produced according to the linguistic validation guidelines of the PedsQL modules. The module was independently translated from English into Italian by two professional bilingual translators. A single, combined version was translated back into English by a local professional bilingual translator native speaker of English. This backward version was compared with the original source version and accepted by the Mapi Research Institute.

### 2.3. Statistical Analyses

Statistical analysis followed the following phases of questionnaire validation: (1) study of the questionnaire’s factorial structure; (2) study of the questionnaire’s internal reliability; (3) study of the questionnaire’s construct validity.

#### 2.3.1. Factorial Structure

For each GCS and CPM subscale, the questionnaire’s factorial structure and dimensionality were evaluated by means of an explorative factor analysis using the principal component analysis method, and the number of dimensions was determined to be equal to the number of eigenvalues greater than 1. We also considered the different scales unidimensionally when the proportion of the variability explained by the first dimension was greater than 50%.

#### 2.3.2. Internal Consistency and Construct Validity

Internal consistency was evaluated by computing Cronbach’s alpha (benchmark value < 0.70) for each subscale. For the GCS, construct validity was evaluated through the known-groups method, studying the differences between healthy participants and CwCP. In known-groups validation, a test is valid when its scores can discriminate across groups that, theoretically, are expected to be different with respect to the construct measured. To evaluate the magnitude of differences, effect sizes were calculated [13] by taking the difference between the healthy sample mean and the mean divided by the healthy sample standard deviation. Effect sizes for different means are classified as small (0.20), medium (0.50), and large (0.80) in magnitude [20]. For the CPM, construct validity was analysed via the associations of the PedsQL^TM^ 3.0 CPM scores with the varying degrees of severity indicated by the GMFCS and MACS levels, employing the one-way analysis of variance. The concordance between patient self-reports and parent proxy-reports was evaluated using the paired *t* test (*p* < 0.005). Statistical analyses were conducted with Statistical Package for Social Sciences (IBM SPSS Statistic, version 24) [21].

## 3. Results

### 3.1. Sample Characteristics

A total of 125 CwCP (80 males (64%); mean age: 8 years (range: 2–18)) and 121 healthy peers (66 males (54%); mean age: 8 years (range: 2–18 years)) fulfilled the inclusion criteria and were enrolled. Of the CwCP group, 60 (48%) children were born at term, 65 (52%) were preterm, and 42 (33.6%) had epilepsy, of whom 11 (8.8%) had drug-resistant epilepsy (Table 1). Both child self-reports and parent proxy-reports were collected in 60 children (48%) aged 5 to 18 years (39 males, 65%), while only parent proxy-reports were collected in the remaining 65 children (52%), according to the administration methods. All responders easily completed the questionnaires and did not demonstrate any difficulty in understanding the items, instructions, and response choices. No missing item responses were found in either the child self-reports or the parent proxy-reports. A total of 57 children were classified as GMFCS level I (45.6%), 25 level II (20%), 14 level III (11.2%), 19 level IV (15.2%), and 10 level V (8%). A total of 32 children were classified as MACS level I (35.6%), 33 level II (36.7%), 8 level III (8.9%), 11 level IV (12.2%), and 6 level V (6.7%). The remaining 35 children were not classified according to MACS because they were younger than 4 years old.

### 3.2. Dimensionality, Internal Consistency, and Construct Validity

Exploratory factor analyses for the GCS and the CPM modules is reported in Table 2. Unlike the GCS, where only a few subscales were unidimensional, all of the CPM subscales proved to be unidimensional, except for Speech and Communication in the child self-reports (Table 2). Cronbach’s alpha was greater than 0.70 for all GCS subscales of the parent proxy-reports, as well as for the Psychosocial Health and Physical Activities subscales of the child self-reports (Table 2). Cronbach’s alpha was greater than 0.70 for all CPM subscales of both parent proxy-reports and—with few exceptions (Fatigue, Speech and Communication)—child self-reports (Table 2). Table 3 reports the construct validity results of the GCS for CwCP and healthy peers. Differences were significant for all scores, showing medium-to-large effect size, except for the child self-report Emotional Functioning score. CPM construct validity data are reported in Table 4a,b. Here, children with higher GMFCS and MACS levels (lower functional abilities) showed significantly lower HRQoL scores across all subscales of the parent proxy-reports and in most subscales of child self-reports of the CPM.

### 3.3. Parent–Child Concordance

In CwCP, child self-report scores were always higher than the proxy-report scores for both modules (Table 5). In the healthy group, the GCS total score and Physical Functioning score were significantly higher in the proxy-reports than in the child self-reports.

## 4. Discussion

The present study confirms the internal consistency and construct validity of the Italian version of the PedsQL^TM^ in CwCP. Although there are other instruments that assess HRQoL in this population, this questionnaire is the only validated HRQoL instrument specific to CwCP covering an age range of 5–18 years for child self-report and 2–18 years for parent proxy-report. In fact, the Child Health Index of Life with Disabilities (CPCHILD) [22] has only been validated for children with severe CP (GMFCS levels IV and V) and provides only a caregiver questionnaire, not a child self-report. Moreover, the KIDSCREEN [23] is not specific to CwCP and does not allow for longitudinal assessment.

In our study, we found that the construct validity and internal consistency of the item and subscales were also maintained in an Italian population of CwCP. At the time of data collection, the two enrolling centres of Milan and Rome were the referral centres for CP in Italy (the former for northern Italy and the latter for central and southern Italy). Thus, we do believe that the sample was representative of the paediatric population with CP. Furthermore, we found that the GCS showed good internal consistency for parent proxy-reports, while some of children’s self-report subscales, such as Social Functioning and Emotional Functioning, failed to reach the benchmark value, which is in line with the results reported by Varni and colleagues [13]. As the authors argue, subscales that do not reach the benchmark value should be used only for descriptive analyses. However, when all the scores from both the parent proxy-reports and the child self-reports were included, internal consistency improved in all subscales except for Emotional Functioning, which remained slightly below the threshold. The potential effect of cognitive impairment in some of these children on the accuracy of their self-reports may have influenced their assessment. In addition, it could be more difficult (and even more unpleasant) for children to express their emotions than adults. This is probably one of the reasons why disease-specific symptoms collected in the CPM showed higher internal consistency. The CPM internal consistency was good in all subscales of parents’ proxy-reports and in most subscales of children’s self-reports, where only Fatigue and Speech and Communications had alpha values below the threshold (Table 2). However, similar results were also found for Fatigue in the original English version and for Fatigue and Speech and Communications in the Chinese version [24]. These results also imply that both GCS and CPM modules could be used for CwCP to assess many aspects of their HRQoL but that more specific aspects, with better psychometric properties, are provided using the CPM [25]. Further work should be conducted to refine the selected items within the subscales with suboptimal internal consistency.

In contrast to the “Study of PARtecipation of children with Cerebral palsy Living in Europe” (SPARCLE) [26], which reported that CwCP experience similar QoL to peers with typical development despite their significant functional limitations [27], in our sample, CwCP scored significantly lower than their healthy peers on the GCS, with the only exception being in the Emotional Functioning subscale. Although there are limitations in interpreting these results, particularly with regard to the Emotional Functioning subscale, as discussed above, they may nonetheless be in line with what others have observed, namely, that the physical well-being scores are lower than the psychosocial well-being in children with disabilities [27]. We found a significant association between physical disability and HRQoL, with children with higher functional abilities showing higher HRQoL. These data partly contradict some previous studies [4,28], highlighting not only the heterogeneous properties of the questionnaires used but also the complexity of the HRQoL construct, now widely described as multidimensional [29]. The PedsQL instrument, particularly the CPM, provides an assessment of HRQoL in CwCP related primarily to child autonomy, the influence of which on general well-being, general aspects of participation, and emotional dimensions certainly warrants targeted investigation. However, given the fundamental role of rehabilitation in promoting and increasing autonomy in daily life, the PedsQL questionnaire may be an appropriate instrument by which to assess the effectiveness of goals-of-care interventions. Interestingly, in the CwCP sample, parents rated their children’s HRQoL lower than did their children themselves in all domains of both modules, in agreement with several previous studies on QoL [3].

Controversy exists as to the factors that influence the degree of agreement between parent and child ratings; a similar pattern of response has also been reported in children with other chronic conditions [27]. By contrast, for healthy children, parents often rate their children’s well-being higher than their children do [30]. Our study seems to confirm these findings: the parents of children with disabilities may not adequately value overall adjustment to their condition, and in contrast, the parents of healthy peers tend to rate their children’s QoL higher than their children rate themselves. Although paediatric patients’ self-reports should be considered the standard for measuring perceived HRQoL, there may be circumstances in which the child is too young, too cognitively impaired, or too ill to complete an instrument. In such cases, a parent proxy-report may be necessary. It is noteworthy that the differences between parent proxy-reports and child self-reports were not significant in some of the CPM subscales, such as Daily Activities, Pain and Hurt, Eating Activities, Speech and Communication. This could support the hypothesis that parents are reliable proxy respondents for children with disabilities who are unable to provide their perspective directly, and that both perspectives should be considered in clinical practice when determining the health and social service needs of patients and their families. Indeed, a child’s underestimated HRQoL from the parents’ point of view may have an impact on disability awareness and, consequently, on clinical care. This impacts treatment planning, which should be individualised, multidisciplinary, and focused on the improvement of daily life.

Consequently, one of the limitations of our study is that the PedsQL presents direct questions, which children sometimes struggle to answer, and which often require the mediation of their parents. Indeed, the questions are always asked looking for negative aspects (e.g., how much pain, how much effort, etc.), leaving out the possibility of the child expressing their experiences more freely.

In reference to this, the SOLE questionnaire [31] seems to have achieved better compliance using neutral illustrations rather than written questions, especially in facilitating the responses of younger children or those with a severe impairment. Another limitation of our study is that children with severe neurofunctional limitations represented a small percentage of the whole sample. This is mainly due to the inclusion criteria and the difficulty faced in completing self-reports for the most impaired children. However, as a trend of HRQoL decline was observed with decreasing functional abilities, we can state that our results could be generalised to all CwCP with a different degrees of severity. Finally, additional limitations of our study include the small sample size and the exclusion of children with severe comorbidities. Nevertheless, for this study, it was necessary to obtain a heterogeneous sample of CwCP for the validation of PedsQL^TM^.

In conclusion, we believe this study is important because it is the first to report the construct validity and internal consistency of the GCS and CPM of the PedsQL in an Italian population of CwCP. Furthermore, this study reports differences in concordance between child self-reports and parent proxy-reports, underlining the importance of using a family-centred instrument to assess the HRQoL. Moreover, the possibility of using a feasible, reliable, and validated HRQoL questionnaire for Italian CwCP can be useful in guiding and evaluating the impact of health services and clinical practice, and in assessing the effect of new treatment strategies on the overall well-being of these patients and their families.

Despite this, we believe that future studies should validate further instruments that investigate in more depth the emotional construct of the child and their family, correlated with QoL, to obtain information about the whole family, as well as to facilitate specific psychological support.

Furthermore, we believe that it is important for future studies to focus on the longitudinal assessment of HRQoL in larger sample, also encompassing children with severe disabilities, and including the assessment of functional scales related to communication (Communication Function Classification System; CFCS) [32] and vision (Visual Function Classification System; VFCS) [33].

## Figures and Tables

**Table 1:** Demographic characteristics of children with cerebral palsy and the healthy sample.

	CwCP	Healthy Sample
Total n	125	121
Mean age (SD)	8 (4.8)	8.4 (4.4)
Age range	2–18	2–18
Male (%)	80 (64%)	66 (54.5%)
Born at term (%)	60 (48%)	-
Born preterm (%)	65 (52%)	-

CwCP, children with cerebral palsy; SD, standard deviation.

**Table 2:** PedsQL^TM^ 4.0 Generic Core Scales and Cerebral Palsy Module Scales dimensionality results.

	n	Items, n	% of Variability Explained by the First Dimension	Number of Eigenvalues > 1	Cronbach’s Alpha
Generic Core Scales Child Self-report					
				
Total Score	60	23	22	7	0.83
Physical Functioning	60	8	45	2	0.79
Psychosocial Health	60	15	25	6	0.79
Emotional Functioning	60	5	42	1	0.61
Social Functioning	60	5	38	2	0.58
School Functioning	60	5	41	2	0.61
				
**Parent Proxy-report** **Total Score**	125	23	34	5	0.91
Physical Functioning	125	8	55	1	0.86
Psychosocial Health	125	15	37	4	0.88
Emotional Functioning	125	5	48	1	0.71
Social Functioning	125	5	49	2	0.74
School Functioning	125	5	56	1	0.80
**Cerebral Palsy Module Scales** **Child Self-report**					
Daily Activities	60	9	66	1	0.93
School Activities	60	4	67	1	0.83
Movement and Balance	60	5	57	1	0.80
Pain and Hurt	60	4	71	1	0.85
Fatigue	60	4	50	1	0.60
Eating Activities	60	5	58	1	0.75
Speech and Communication	60	4	47	2	0.63
**Parent Proxy-report**					
Daily Activities	125	9	73	1	0.95
School Activities	125	4	78	1	0.91
Movement and Balance	125	5	59	1	0.83
Pain and Hurt	125	4	74	1	0.87
Fatigue	125	4	76	1	0.89
Eating Activities	125	5	71	1	0.88
Speech and Communication	125	4	84	1	0.93

**Table 3:** Scale descriptives for the PedsQL^TM^ 4.0 Generic Core Scales for the parent proxy-reports and the child self-reports, as well as comparisons between paediatric CP patients and healthy children scores.

	Items, n	CwCP, n	Mean (SD)	Healthy, n	Mean (SD)	Difference	Effect Size
Parent Proxy-report							
Total Score	23	125	63.8 (18.6)	121	86.5 (9.3)	22.69 **	2.44
Physical Functioning	8	125	55.6 (28.7)	121	90.5 (10.3)	34.87 **	3.37
Psychosocial Health	15	125	68.5 (17.3)	121	84.2 (10.4)	15.70 **	1.51
Emotional Functioning	5	125	69.8 (19.6)	121	76.9 (14)	7.14 *	0.51
Social Functioning	5	125	67.8 (19.9)	121	90.5 (11.9)	22.69 **	1.91
School Functioning	5	114	66.9 (25.3)	115	85.7 (14.2)	18.84 **	1.32
**Child Self-report**							
Total Score	23	60	71.8 (14.2)	88	83.1 (9)	11.31 **	1.26
Physical Functioning	8	60	68.4 (23.2)	88	86.8 (9.4)	18.37 **	1.95
Psychosocial Health	15	60	73.7 (14.5)	88	81.1 (10.2)	7.45 **	0.73
Emotional Functioning	5	60	72.1 (19.1)	88	72.6 (15.7)	0.50	0.32
Social Functioning	5	60	74.8 (17.5)	88	88.7 (11.6)	13.87 **	1.20
School Functioning	5	59	73.7 (18.6)	88	82.1 (13.5)	8.36 *	0.62

CwCP, children with cerebral palsy; SD, standard deviation. *t*-test significance: ** *p* < 0.001; * *p* < 0.05. Effect sizes are designated as small (0.20), medium (0.50), and large (0.80).

**Table 4:** (**a**) PedsQL^TM^ CPM scores by GMFCS levels. (**b**) PedsQL^TM^ CPM scores by MACS levels.

(a)								
	GMFCS							
CPM	Level I	Level II	Level III	Level IV	Level V	*p*	df	F
	Mean (SD)							
**Child Self-report**	n = 33	n = 12	n = 7	n = 5	n = 3			
Daily Activities	85.1 (23.4)	69 (29.1)	46 (23.3)	11.6 (9.7)	10.2 (17.6)	<0.001	4.59	17.23
School Activities	86.7 (22.6)	87.5 (19.4)	72.3 (27.9)	46.3 (28.5)	25 (43.3)	<0.001	4.59	7.39
Movement and Balance	83.6 (17.3)	82.1 (18.4)	53.6 (23.4)	24 (22.2)	41.7 (27.5)	<0.001	4.59	15.29
Pain and Hurt	86 (20.6)	75.5 (29.4)	83.9 (20)	81.3 (25.8)	66.7 (19.1)	NS	4.59	0.84
Fatigue	74.4 (20.9)	70.3 (24.6)	89.3 (12.9)	63.8 (10.3)	72.9 (13)	NS	4.59	1.43
Eating Activities	95.5 (9.4)	93.8 (12.5)	77.7 (34.2)	81.3 (17.7)	66.7 (57.7)	NS	4.59	2.98
Speech and Communication	92.2 (11.4)	83.9 (19.5)	82.1 (24.6)	81.3 (25.8)	87.5 (12.5)	NS	4.59	1.17
**Parent Proxy-report**	n = 57	n = 25	n = 14	n = 19	n = 10			
Daily Activities	67 (30.3)	46.2 (28)	29.7 (23.4)	6.4 (14.3)	4.8 (10.2)	<0.001	4.124	27.55
* School Activities	80.8 (23.6)	60.4 (27.8)	57.8 (35.9)	23.4 (28.8)	8.9 (23.6)	<0.001	4.89	18.64
Movement and Balance	71 (21)	61.4 (20.3)	38.9 (21.3)	19.5 (23.9)	18 (18.7)	<0.001	4.124	31.42
Pain and Hurt	85.5 (16.7)	82.5 (25.3)	88 (14.6)	71.7 (28.7)	51.9 (27.3)	<0.001	4.124	6.51
Fatigue	75.9 (22)	61.3 (29)	75.5 (24.3)	61.5 (32.2)	54.4 (36.9)	0.03	4.124	2.79
Eating Activities	90.2 (17.2)	79.8 (26.5)	82.6 (17.9)	44.1 (37.3)	30 (42.6)	<0.001	4.124	20.12
* Speech and Communication	86.6 (17.9)	70.5 (34)	84.4 (24.1)	43.8 (38.2)	44.6 (45.6)	<0.001	4.89	7.66
(**b**)								
	**MACS**							
**CPM**	**Level I**	**Level II**	**Level III**	**Level IV**	**Level V**	***p***	**df**	**F**
	Mean (SD)							
**Child Self-report**	n = 22	n = 27	n = 5	n = 5				
Daily Activities	83.6 (27.9)	68.6 (29)	52.2 (36.6)	18.9 (27.7)		<0.001	3.58	7.32
School Activities	90.3 (22.4)	80.8 (20.9)	71.3 (28.2)	40 (42.8)		0	3.58	6.10
Movement and Balance	81.4 (20.4)	77.6 (17.1)	60 (36.2)	33 (40.3)		<0.001	3.58	7.14
Pain and Hurt	82.7 (27.5)	84 (19.8)	63.8 (17.9)	93.8 (8.8)		NS	3.58	1.62
Fatigue	81.3 (18.9)	69.7 (21.3)	71.3 (22.4)	73.8 (20.9)		NS	3.58	1.34
Eating Activities	95.5 (11.9)	91.9 (12.4)	82.5 (39.1)	86.3 (19)		NS	3.58	1.11
Speech and Communication	94.3 (12)	85.7 (18.3)	81.3 (15.9)	85 (22.4)		NS	3.58	1.61
**Parent Proxy-report**	n = 32	n = 33	n = 8	n = 11	n = 6			
Daily Activities	75 (25)	54.8 (30.7)	36.1 (31.7)	10.4 (18)	0 (0)	<0.001	4.89	1.36
* School Activities	60.4 (22.3)	62.9 (29.2)	49.2 (33.7)	27.3 (28.5)	0 (0)	<0.001	4.89	1.37
Movement and Balance	72.8 (21.3)	61.1 (21.3)	48.1 (32.3)	22.3 (26.6)	7.5 (8.8)	<0.001	4.89	18.17
Pain and Hurt	84.6 (21.8)	79.2 (20.2)	72.7 (23.1)	84.1 (20)	60.4 (29)	NS	4.89	1.93
Fatigue	79.9 (20)	59.9 (25.7)	62.5 (28.2)	73.9 (32.5)	40.6 (37.6)	0	4.89	4.43
Eating Activities	92.6 (14.8)	80.7 (27.5)	78.9 (24.1)	59.7 (36.1)	0 (0)	<0.001	4.89	20.84
* Speech and Communication	89.1 (15.2)	81.1 (26.1)	65.6 (38.7)	42.6 (33.5)	18.6 (29.3)	<0.001	4.89	14.93

* n = 42, 21, 8, 12, 7. CPM, Cerebral Palsy Module; GMFCS, Gross Motor Function Classification System; MACS, Manual Ability Classification System; SD, standard deviation; df, degree of freedom.

**Table 5:** PedsQL^TM^ 4.0 Generic Core Scales and Cerebral Palsy Module Scales: comparisons between parents and children.

Children			Parents			
Generic Core Scales	n	Mean (SD)	Mean (SD)	*t*-test	df	*p*
Total Score	60	71.7 (14.3)	65.7 (16.6)	−3.77	59	0.000
Physical Functioning	60	68.2 (23.4)	61.3 (26.6)	−2.66	59	0.010
Psychosocial Health	60	73.6 (14.6)	68.1 (15.3)	−2.95	59	0.005
Emotional Functioning	60	72.1 (19.1)	67.8 (19.6)	−1.88	59	0.065
Social Functioning	60	74.8 (17.5)	66.2 (17.6)	−3.67	59	0.001
School Functioning	59	74 (18.7)	70.4 (19.8)	−1.39	57	0.171
**Children**			**Parents**			
**Cerebral Palsy Module Scales**	**n**	**Mean (SD)**	**Mean (SD)**	***t*-test**	**df**	***p***
Daily Activities	60	67.5 (34.4)	63 (32.3)	1.84	59	0.070
School Activities	60	78.8 (28.9)	71.2 (30.5)	2.41	59	0.019
Movement and Balance	60	72.8 (26.8)	60.8 (26.3)	4.70	59	0.000
Pain and Hurt	60	82.3 (22.8)	79.2 (21.5)	1.62	59	0.110
Fatigue	60	74.4 (20.5)	66.2 (23.9)	3.05	59	0.003
Eating Activities	60	90.4 (20.1)	88.1 (22.2)	1.03	59	0.306
Speech and Communication	60	88.2 (16.6)	84.9 (19.6)	1.17	59	0.247

SD, standard deviation; df, degree of freedom.

## Data Availability

The original contributions presented in the study are included in the article, further inquiries can be directed to the corresponding author.
